# Reports of Cases Occurring in the Medical Ward of the Buffalo Hospital

**Published:** 1857-07

**Authors:** A. W. Nichols

**Affiliations:** Assistant to Chair of Clinical Medicine, University of Buffalo


### BUFFALO MEDICAL JOURNAL

AND

### MONTHLY REVIEW.

**VOL. 13.** JULY, 1857. **NO. 2.**

### ORIGINAL COMMUNICATIONS.

ART. I.—*Reports of Cases treated in the Male Medical Ward of the Buffalo Hospital of the Sisters of Charity*, AUSTIN FLINT, M. D., *Attending Physician, during the College Session of* 1856–7, *being from October* 1, 1856 *to February* 18, 1857. Reported by A. W. NICHOLS, M. D., Assistant to Chair of Clinical Medicine, University of Buffalo.

No. IV. *Intercostal Neuralgia; Dorso-intercostal Neuralgia; Pleuralgia. An Address read at the semi-annual meeting of the Medical Society of Erie County, in Buffalo, June 9th*, 1857.

MR. PRESIDENT AND GENTLEMEN OF THE SOCIETY:

Permit me to thank you for the honor you have conferred upon me, in appointing me to address you on this occasion.

The subject which I have selected as an interesting matter of discussion, has already been amply treated of, by MM. Nicod, Bassereau, and Valleix, as well as by a few English and American physicians. I cannot offer you, therefore, anything novel; but will be able, perhaps, to bring before you more explicitly the important features of *Intercostal Neuralgia*.

The following remarks are based upon an analysis of the records of twenty cases of neuralgia, of which fourteen belonged to the disease under consideration, and upon the results of personal dissections of the intercostal nerves.

The conclusions are strikingly corroborative of the results of previous investigations. It was in 1818, that Nicod first gave a description of this disease, which has, within the past twenty years, been more fully treated of by MM. Bassereau and Valleix.

Intercostal neuralgia has been, and is still, to some extent, mistaken for *pleuradynia;* the distinctive signs of these two diseases have not always been recognized; in fact, *pleurodynia* has been the only term employed by many physic'ans to denote the various painful conditions of the parietes of the thorax, which could not be attributed to inflammation. Intercostal neuralgia has also been confounded with *spinal irritation* or *spinal tenderness.* It is our intention to point out the distinctive traits of intercostal neuralgia, and to show how it may be distinguished from pleurodynia or from spinal irritation so-called. These conclusions will be drawn from an analysis of cases recorded in the hospital-books of Prof. Flint, for the past year or more.*

(*a.*) *Causes.* The causes of intercostal neuralgia may be classified as the predisposing and the occasional or exciting.

Of the former or *predisposing,* all of the patients, fourteen in number, were *above the period of puberty;* and only three over thirty-five years of age.

The great number of male patients, thirteen, is due to the fact that these observations were confined to the male ward, with a single exception. The disease, however, is much more frequently met with amongst *females,* especially during the period of life in which menstruation occurs. The general condition of the patient, his *constitution,* in other words, will be found, *not enfeebled* usually, but good. The disease appears to exist more commonly in *persons actively engaged,* than in those pursuing sedentary occupations; seven of these patients were laboring men, and only one was not very actively engaged.

The *hygienic conditions* under which these patients were placed, (such as food, clothing, residence,) were *only fair,* not good.

The greater frequency of this disease during some *seasons of the year* than others, is striking; all but two of the cases occurred during the latter part of winter and the early part of spring. The changeable nature of this

---

* It should be remembered, that the number of cases of neuralgia recorded, does not furnish any evidence of the frequency of these diseases; for, as a general rule only the most striking cases were noted. N.

latitude, at these particular periods, is well known; probably, *atmospheric changes*, then, exert some influence as one of the predisposing causes of intercostal neuralgia.

The *left side* is more commonly affected than the right; and when the disease is manifested on both sides, the pain is more severe on the left; of these fourteen cases, the left side was affected in eight cases, and both sides in one case. The pain is not usually found to exist in every intercostal space, but is more frequently present, and to a greater degree, in the 5th, 6th, 7th, and 8th interspaces.

The exciting causes may be various; some times *long continued and active exertion* of either upper extremity may give rise to this disease, as occurred in one of these cases; a young man used for some weeks, his right arm almost exclusively in his business; the right side was attacked. A *blow on the chest* may likewise produce the disease; one of these patients received a blow from a fall on the affected side.

It appears sometimes during or immediately after *chronic cutaneous diseases*, particularly when the eruption is most copious on the chest or confined to that portion of the body; as, after or during herpes zoster or zona. Valleix has observed it frequently to accompany or follow *capillary bronchitis.*

The diseases, during or after the course of which intercostal neuralgia is most commonly met with, in this climate, are *pulmonary tuberculosis* and *periodical fever;* in two of these cases, it accompanied the former disease, and in six, it accompanied or followed the latter disease. Various forms of *neuralgia* may exist previously in other parts of the body, and often accompany or follow the disease; *gastralgia* is particularly cited by writers, as one of the most troublesome attendants of intercostal neuralgia. In the female case, there existed previously a notable cough, which resembled the "barking of a dog;" it was a hysterical cough.

In two of the cases, *colica pictonum* or *saturnina*, existed; the pain, in both these instances, was acute and lancinating, and persistent.

(*b.*) *Anatomy.* In order to comprehend fully the nature and importance of some of the signs of intercostal neuralgia, it is necessary to enter somewhat in detail, into the anatomical peculiarities of this portion of the nervous structure.

The intercostal nerves are *twelve* in inumber: they are essentially the *anterior divisions* of the *dorsal* nerves. Every spinal nerve springs from the spinal cord by two roots, which uniting in the intervertebral foramen, divide into two portions; one of these being directed *backward*, supplies the skin and muscles adjacent to the spinous processes; and the other passes *forward*, and is distributed to the integuments and muscles of the lateral and anterior portions of the trunk; the latter divisions of the spinal nerves communicate freely with the sympathetic nerve. In the *dorsal* region, the *posterior* division of these nerves are much smaller than the anterior divisions; and they soon divide into branches supplying the muscles called *internal*, and those going to the integument called *external;* this separation takes place between the transverse processes of the vertebræ. On the other hand, the *anterior* divisions of the dorsal nerves, may be divided equally, into those which are confined to the thoracic parietes, and into those which are continued forward to the muscles and integument of the abdomen. The former, six in number, may be termed *upper intercostal* nerves; and the latter, six in number, *lower intercostal* nerves. They are called *intercostal*, on account of their relations with the ribs; they are distributed to the muscles and integument of the thorax and abdomen; they are composed of *motor* and *sensory* filaments; and they communicate with the ganglia of the sympathetic nerve, in the vertical line through the heads of the ribs. *The upper intercostal* nerves pass between the two layers of intercostal muscles, and about midway between the spinous processes of the vertebræ and edge of the sternum, give off their *first* branches which, from their position and from their distribution, are called *lateral cutaneous* branches; the main trunks of the nerves penetrating the internal intercostal muscles, continue forward, amid their fibres as far as the costal cartilages; here they are in contact with the pleura; near the margin of the sternum they pierce the *intercostal* and *pectoralis major* muscles; and instead of communicating with their fellows on the opposite side of the chest, run *backward* between the layers of superficial fascia covering the great pectoral muscle; and *terminate* by filaments to the integument, and by communicating branches with the lateral cutaneous branches just mentioned. These lateral cutaneous branches, after penetrating the external intercostal and the serratus magnus muscles, in that portion of the lateral surface which lies between the converging fibres of the latissimus dorsi and pectoralis major muscles, divide into two sets of branches, one passing *forward* and communicating, as stated, with the terminal filaments of the main trunks; the other passing *backward* in the layers of the superficial fascia investing the latissimus dorsi, and are distributed to that muscle and to the integument over the scapula.

The *lower* intercostal nerves, likewise six in number, pass through the intercostal spaces; the lowest of this set having no rib below it. From the anterior terminations of the intercostal spaces, the nerves are continued forward, between the obliquus internus and transversalis muscles of the abdodomen, tr the outer edge of the sheath of the rectus abdominis; they perforate this sheath and are distributed in part to the rectus; and the *terminal filaments* penetrating again the sheath, near the median line of the body pass *backward* as cutaneous branches, in a similar manner to that of the terminal filaments of the upper intercostal nerves. The main trunks likewise give off *lateral cutaneous* branches about the middle of their course, which are divided and distributed in a manner similar to that of the lateral branches of the upper intercostal nerves.

In this anatomical description, there are several points having a bearing on the diagnostic signs of this form of neuralgia, which it is necessary to recollect. In the first place, there is *no difference* in the course or distribution of these nerves on either side to account for the *greater frequency* of the disease on the *left side* of the chest. Next, these nerves *do not form any communication* with their fellows of the opposite side of the thorax, so far as our dissections have informed us. Again, they *do not communicate freely with each other* on the same side; they *do not form any plexus* by the interlacement of their fibres; in this respect, differing remarkably from the other spinal nerves. Lastly, they *give off very few branches*, and *these in a particular locality;* these branches pass through muscles or their tendinous expansions, to the integument; the perforation in the tendinous structures being *invariably more or less quadrilateral;* and there are *three situations* at which, the *dorsal spinal nerves* penetrating muscular fibres, become *subcutaneous*, *one to the side of the spinous processes*, *one about midway from these processes* to *the sternum*, *and one close to the margin of the latter bone or to the median line of the body below the sternum.*

(*c.*) *Symptoms.* Intercostal neuralgia, it has been observed, may manifest itself during the course of diseases of the thoracic or abdominal viscera. But physical exploration of the chest would inform us of the nature and extent of a morbid condition of any of the thoracic viscera; and the signs presented in diseases of the abdominal viscera, could not usually fail to lead to a detection of the locality or character of the morbid state. Thus, intercostal neuralgia accompanies pulmonary tuberculosis and capillary bronchitis; and gastralgia has been found to be persistent, especially when intercostal neuralgia also exists. However, when this disease is uncomplicated, the symptoms presented are *remarkably characteristic.* The patient may be attacked suddenly with sharp and lancinating pain; but, more commonly, a

*dull pain*, intermittent or continuous, or merely a sense of uneasiness, not of actual pain, exists for a few days. The pain sooner or later *becomes acute;* it may be lancinating; and if so, the painful sensations are experienced in the course of the branches of the intercostal nerves. It may be intermittent, but generally is *continuous;* it was intermittent in three of these cases, and continuous in eleven. If the pain be intermittent, it does not usually disappear entirely; if so, it may be readily *elicited by pressure;* neither does the pain remain equally intense, if it be continuous; it may increase or diminish in intensity.

The most remarkable feature of this pain, is that it is usually *confined to particular points* on the thoracic or abdominal parietes; these may be *one, two or three in number;* generally, more than one painful point is present. If the pain be lancinating, it will shoot from these points along the course of the nerves. If pain is already manifested at these situations, or if it be intermittent, when *pressure* is made over these particular localities, the pain is very much *increased in intensity*, or is *suddenly elicited.* Where are these points situated?

The *first* or *vertebral* point is *slightly* to the outside of the spinous processes of the dorsal vertebræ and almost over the dorsal-intervertebral foramina, just opposite the divisions of the spinal nerves into their anterior and posterior branches.

The *second* or *lateral* point is *about midway* between the spinous processes and the margin of the sternum.

The *third* or *sternal* or *epigastric* point is—in the thorax—near to the edge of the sternum,—in the abdomen—just to the side of the median line.

The pain is *limited to these points;* it is *very circumscribed;* it extends over a surface which may be *covered by the ball of the thumb*, or about ⅝ of an inch in diameter. If the pressure be continued, that is, not immediately removed, the pain very commonly disappears momentarily. That this pain is not produced by the efforts of pressure, that is, not due to the degree of pressure exerted, is shown by employing the same or a greater amount of pressure on the unaffected side, *without the manifestation of pain*, or discomfort even. Besides the pain being elicited or increased by pressure, *acts of coughing* or *forced inspirations*, or even *movements of the arms or trunk*, give rise to the pain or augment it.

(*d.*) *Frequency: Duration: and Termination.* Intercostal neuralgia is much more common than *sciatica:* of nineteen recorded cases of neuralgia, only two were of sciatica, and fourteen of intercostal neuralgia. It is *much more frequently observed than pleurodynia:* during the same period that the fourteen cases of intercostal neuralgia occurred, *only a single case of pleurodynia* was noted; this was made one of the circumstances for investigation during the collection of these cases.

This disease is *apt to become chronic*, and to prove difficult of perfect relief. Six of these fourteen cases continued for some weeks before marked relief was obtained. As the attack may be sudden in a few cases, *so may its termination be;* this occurred in a single instance, one of the patients affected with pulmonary tuberculosis, died.

(*e.*) *Diagnosis.* After what has been remarked under the *anatomy* and *symptoms* of this disease, it will be comparatively easy to distinguish intercostal neuralgia from thoracic or abdominal disease, or from affections of the spinal cord or vertebræ themselves, or even from other forms of neuralgia. From other neuratic affections, it may be known by the characteristic features of the pain in intercostal neuralgia. These same characteristics would serve to assist in the diagnosis of caries of the vertebræ, or in irritation, inflammation, or softening of the medulla spinalis. And in abdominal disease, (as splenitis, gastritis, or peritonitis,) these signs would likewise aid us.

A careful examination of the chest, by the various methods of physical exploration, would almost always render the diagnosis of thoracic-visceral disease, certain. But, there are *two thoracic affections*, the distinctive signs of which — in reference to the disease under consideration — it would be well to mention more particularly. In connection with some cases of pulmonary tuberculosis, an inflammation of the pleura, circumscribed and with no effusion, occurs: it is called *dry pleuritis;* also, in the very earliest stages of *acute pleuritis*, before effusion has taken place; in either of these conditions the importance of an acquaintance with the characteristic pain of intercostal neuralgia, is apparent; besides the different nature of the pain, in these inflammatory diseases, there is more or less difficulty of respiration, and febrile movement, and there may be partially suppressed cough and friction-sounds. Moreover, the pain in inflammatory affections of the thoracic viscera, is supposed to be *increased by forced breathing* more than by the movements of the arms or trunk; whilst the reverse is generally the case in neuralgic disease.

For a rheumatic affection, however, intercostal neuralgia is most frequently mistaken; this is *pleurodynia* or rheumatism of the intercostal muscles. It is only of late years, that the distinction between these two diseases, has been drawn, and now they may be easily and certainly distinguished from each other. Valleix says, "that when the pain may be attributed to muscular rheumatism, it is *more diffused, not circumscribed.* The pain on pressure, is generally *less acute*, and is *not confined to particular points* on the chest. In no case is there observed *pain seated in two points exclusively*, and these situated a greater or less distance from each other, as is the case in intercostal neuralgia. When the rheumatism is severe, the pain is *less intense on pressure*, than during movements of the body and during acts of coughing. As regards the lancinating pains, their distinctive characters are not well defined; and this explains how we may commit many errors, in neglecting to make a direct examination of the chest."

A tabular view of the distinctive signs of *intercostal neuralgia* and of *pleurodynia*, is here appended:

*Diagnostic Signs of*

| *Intercostal Neuralgia.* | *Pleurodynia.* |
|---|---|
| 1. Painful points generally *slightly diffused* or circumscribed. | 1. Pain occupying a surface more extended, *not circumscribed.* |
| 2. Painful points *separated* to a greater or less distance from each other. | 2. Pain *not confined to points* more or less remote from each other. |
| 3. Pain generally *more acute* on pressure. | 3. Pain generally *less acute* on pressure. |
| 4. Pain *less intense* on *motion* of the trunk or arms. | 4. Pain *more intense*, sometimes insupportable *on motion* of the trunk or arms. |

(*f.*) *Treatment.* The treatment may consist of external or of internal remedies. The *external* applications employed, have been *blisters* and *sinapisms;* in both the cases in which *blisters* were used, much relief was obtained, but the pain did not entirely disappear. The French writers place much reliance on small and often repeated vesications in the cure of this and other forms of neuralgia. *Sinapisms* were employed in six cases, with temporary benefit in each case, their effects are not permanent. The *internal* remedies consisted of *anodynes* and of *tonics:* the *extract of belladonna*, in doses of $\frac{1}{4}$ to $\frac{1}{8}$ of a grain, sometimes produce *much relief;* the *sulphate of morphia* was given in six cases and always with striking beneficial results. The quantity that may be given, under these circumstances, is very large: doses *of a grain*, repeated every two or three hours, were administered without any unpleasant effects: the usual dose, however, did not exceed *half a grain.* The intensity of the pain is some measure of the amount required to produce relief.

The *tonics* employed in these fourteen cases of intercostal neuralgia, were the *sulphate of quinia* and the *citrate of iron and quinia:* the *former* employed in doses of five grains, three times daily, and continued for a week or more, *always gave relief;* and the pain disappeared and did not return: this occurred in *ten* of the cases under consideration.

